# Sixty-Fifth Annual Meeting of the New York State Medical Society

**Published:** 1871-02

**Authors:** 


### Editorial.

### Sixty-Fifth Annual Meeting of the New York State Medical Society.

The recent meeting of the State Medical Society, which opened in the City Hall, at Albany, on the 17th inst., was one which was well attended, and which was of much interest and value to the members there present. After calling of the meeting to order, and after prayer was offered by the Rev. Dr. Clark, the President of the Society, Dr. S. O. Vanderpoel, of Albany, made his Inaugural Address, in which he recommended the repeal of the regulation forbidding the publication, in the "Transactions," of articles which have previously been in print; the continuance of the work of medical registration; collegiate instruction in mental diseases; proper recognition of labors at the Surgeon General's Office; and appropriate tributes to the memory of illustrious members whose deaths have occurred the last year. Among the names of those present at the last meeting, we are happy to notice those of Prof. T. F. Rochester, Dr. C. C. Wyckoff, and Dr. C. C. F. Gay, of this city. The following appointments, with reference to committees, are among those made: on Arrangements, Drs. Quackenbush, W. H. Bailey and J. V. Kendall; on credentials, Dr. A. N. Bell; on invitations to the Governor and Legislature, Dr. J. V. Cobb; on publication, Dr. Hutchinson; on psychology, Dr. J. P. Gray; on microscopical labors in the Surgeon General's Office, Dr. E. R. Hun; on business, Dr. W. C. Wey; and on nominations, Dr. T. F. Rochester for the eighth district.

Dr. Vanderpoel, the President, extended an invitation to the Society to enjoy his hospitalities on the evening of the second day of the meeting. This invitation was accepted, with the thanks of the Society. On the occasion of the first evening session of the Society, Dr. H. D. Noyes, of New York City, delivered a lecture on the "Theory of Vision," which was finely illustrated by means of the oxycalcium light, and a large canvas screen, together with various models. The subjects of vision, accommodation and astigmatism, were very satisfactorily presented before a large and appreciative audience.

The papers read before the Society were:—Laceration of perineum and bladder, by Dr. Burr, of Binghampton; two cases of Luxation of the elbow backwards, by Dr. Sayre; Puerperal convulsions, by Dr. Jewett; Urethrocele, catarrh and ulceration of the Female Bladder, by Dr. Bozeman, of New York City; Relations of insanity to physical diseases, by Dr. J. P. Gray; Operations for divergent squint, by Dr. Agnew; Anaesthesia and an ether apparatus, by Dr. E. R. Squibb; Spinal irritation, by Dr. Samuel Peters, of Cohoes; Glosso-pharyngeal paralysis, by Dr. E. R. Hun, of Albany; Insanity, by Dr. Peters; Pepsin medicines, by Dr. Hawley; Statistical report of four hundred and ninety-four cases of aural diseases, by Dr. Roosa; Prolapsus uteri, its cause and treatment, by Dr. Thomas Addis Emmet; Early diagnosis of pulmonary phthisis by the microscope, by Dr. Joseph G. Richardson, of Pennsylvania Hospital—the latter was read by invitation.

The following papers were read by title, and referred to committee on publication: Absorption of bone, a case by Dr. J. N. Mead; Inoculation with tubercular matter, by Dr. L. Norton; Case of congenital hypertrophy of the tongue, by Dr. Wm. Vosburgh; Contagion, by. Dr. S. Mosher; case of Ovariotomy, by Dr. J. V. P. Quackenbush; case of Inversion of the uterus and its reduction, by Dr. J. V. P. Quackenbush; Trismus nascentium, by Dr. J. S. Bailey; One of the modes of death from chloroform, by Dr. A. H. Smith; Radical cure of Hernia, also Ligature of subclavian artery for aneurism, by Dr. C. C. F. Gay, of Buffalo; Fracture of Vertebra, by Dr. P. O. Williams; Obituary notices of Dr. Post and Hasbrouch.

Dr, T. F. Rochester, chairman of committee on prize essays, reported that the "Merritt H. Cash prize" was awarded to Dr. S. Fleet Speir, of Brooklyn, for a paper entitled "A new method of arresting surgical hemorrhage by the artery constrictor." The Corliss prize essay was that by Dr. Ghislani Durant, of New York. Reports were presented from the State Medical Societies of Maine, Vermont, Massachusetts and Rhode Island.

A resolution of apology to Dr. Parks, of London, for plagiarism in the volume of transactions for 1868, was passed. Protests from members of the Fifth district, from the Clinton and Dutchess County Societies, against the appointment of permanent members, as from districts to which they do not belong, were presented. On motion by Dr. Jacobi, the following were appointed, by the President, a committee to inquire into the subject of Infant Mortality, viz: Drs. Jacobi, J. P. White, H. W. Dean, Thos. Hun and J. C. Hutchinson. Dr. O. White, of the committee on by-laws, reported that the St. Lawrence, Monroe, Genesee, Cattaraugus, and Chautauqua County Societies, had submitted their constitutions and by-laws to that committee. By vote of the Society its by-laws were amended, so that they now allow papers which previously have been in print, to, nevertheless, be published in the transactions; also, that papers read before the Society may be published by the author, except the Society object; and papers whose titles simply are read, are subject only to objection by the committee on publication. A vote of thanks was rendered Dr. Sayre for his successful efforts in securing a judicial decision which established a legal principle of great value to the whole medical profession. Resolutions were also passed with respect to instructions in nervous diseases; for the printing of three hundred extra copies of the proceedings of the Society; on Vaccination as a condition of admission to public schools; of appreciation of labors at the Surgeon General's Office. A resolution of sympathy with the N. Y. College of Pharmacy, and endorsement of its petition for $10,000 aid from the State, was proposed by Dr. E. R. Squibb, and after some discussion was withdrawn for want of time for proper consideration of the matter.

On motion by Dr. E. Eliott, the Society passed a resolution requesting that the bill now before the Legislature, to secure physicians in the payment of costs and damages in suits for malpractice, by compelling the plaintiff to give satisfactory bonds before the commencement of any suit, be enacted, as being a matter of the highest interest to every physician.

Dr. T. H. Squire, of Elmira, exhibited to the Society a "Vertebrated Prostatic Catheter." Dr. Sayre presented a "Jointed Silver Probe" for exploring long and tortuous sinuses. Dr. S. Fleet Speir, of Brooklyn, exhibited an "Artery Constrictor," for restraining hemorrhage by invagination of the internal and middle coats of arteries.

The officers of the Society appointed for the ensuing year are:—President, Dr. William C. Wey, of Elmira; Vice-President, Dr. Andrew F. Doolittle, of Herkmer; Secretary, William H. Bailey, of Albany; Treasurer, Charles H. Porter, of Albany. The censors for the Western district are Drs. J. F. Miner and C. C. Wyckoff, of Buffalo; and D. Calvin, of Clyde. For committee on correspondence, Dr. H. N. Eastman, of Geneva, was elected for the Seventh district, and Dr. J. F. Miner for the eighth. Dr. James P. White, of Buffalo, was elected one of the delegates to the American Medical Association. The committee on prize essays consists of Drs. T. F. Rochester and Sandford Eastman, of Buffalo, and H. W. Dean of Rochester. Dr. Thomas Hun, of Albany, is Chairman of the Committee on Publication; and Dr. Oliver White, of New York, of that on By-Laws.

The President's address was upon the subject of the "Study of Pathology," and it is said to have been of a superior and masterly character. At the President's reception, which was held at his residence immediately after the delivery of the address, there were present many eminent executive and judiciary officers, as well as members of the press. Dr. Vanderpoel did honor to the Society in the rare and generous hospitalities which were richly enjoyed.

For assistance in the preparation of this report, we present our acknowledgments to DR. WILLIAM H. BAILEY, of Albany; DR. T. F. ROCHESTER of this city; the ALBANY EXPRESS, ARGUS & JOURNAL, and the NEW YORK MEDICAL RECORD.

